# Association of serum lutein and zeaxanthin with quantitative measures of retinal vascular parameters

**DOI:** 10.1371/journal.pone.0203868

**Published:** 2018-09-27

**Authors:** Neelam Kumari, Joanna Cher, Edwin Chua, Haslina Hamzah, Tien Yin Wong, Carol Y. Cheung

**Affiliations:** 1 Department of Ophthalmology and Visual Sciences, Khoo Teck Puat Hospital, Singapore, Republic of Singapore; 2 Singapore Eye Research Institute, Singapore National Eye Center, Singapore, Republic of Singapore; 3 Ophthalmology and Visual Sciences Academic Clinical Programme, Duke-NUS Graduate Medical School, National University of Singapore, Singapore, Republic of Singapore; 4 Department of Ophthalmology and Visual Sciences, the Chinese University of Hong Kong, Shatin, Hong Kong, The People’s Republic of China; The Pennsylvania State University, UNITED STATES

## Abstract

To evaluate the association between serum carotenoids and quantitative measures of retinal vasculature in elderly Singapore Chinese subjects. The following details were collected in 128 healthy subjects: sociodemographics, lifestyle information, medical and drug history, and anthropometric measurements. Serum concentrations of carotenoids were estimated in fasting venous blood using high performance liquid chromatography. Retinal vascular parameters were quantitatively measured from retinal photographs using a computer-assisted program (Singapore I Vessel Assessment). The mean age of the population was 54.1 years (range 40 to 81 years). In multiple linear regression analysis, per SD decrease in retinal arteriolar caliber [β = 0.045 (0.003 to 0.086), p = 0.036], per SD increase in retinal venular caliber [β = -0.045 (-0.086 to -0.003), p = 0.036] and per SD increase in arteriolar branching angle [β = -0.039 (-0.072 to -0.006), p = 0.021] were associated with decreased serum lutein. Per SD increase in retinal venular tortuosity [β = -0.0075 (-0.0145 to -0.0004), p = 0.039] and per SD increase in arteriolar branching angle (β = -0.0073 [-0.0142 to -0.0059], p = 0.041) were associated with decreased serum zeaxanthin. None of the other carotenoids demonstrated meaningful relationship with quantitative measures of retinal vasculature. Lower levels of lutein and zeaxanthin demonstrated significant relationship with adverse quantitative measures of retinal vasculature in elderly healthy subjects.

## Introduction

The retinal vessels are accessible for non-invasive visualization, and therefore, provide a means to study early structural changes and pathologic features of the human microcirculation. Over the last decade, large-population based studies have shown that retinal vascular parameters are associated with a wide range of subclinical (atherosclerosis) and clinical cardiovascular diseases (hypertension, heart diseases and stroke) [1–3]. Moreover, studies have also evaluated the link between retinal vascular caliber and ocular diseases, such as diabetic retinopathy [4] retinal vein occlusion [5] and age-related macular degeneration [6]. Alterations in retinal vasculature may thus reflect a state of vascular dysfunction and might potentially predict onset of systemic and ocular disease development.

It has been postulated that retinal vascular parameter may reflect the cumulative structural vascular damage from multiple factors. These include demographic factors (age, gender, ethnicity), environmental and lifestyle factors (smoking, obesity), physiological blood flow factors (oxygenation, shear stress) and genetic factors [7]. Recently, a study by Daien et al. demonstrated that narrower retinal arteriolar caliber was independently associated with lower glutathione peroxidase activity (endogenous antioxidant defense system) in a community dwelling cohort thereby suggesting that retinal vascular parameters may also be influenced by oxidative stress [8].

Carotenoids by nature of their biochemical structure and function help neutralize reactive oxygen species and prevent oxidative stress (biological antioxidants) [9]. Lutein (L) and zeaxanthin (Z) are the two carotenoids that are selectively accumulated in the primate macula to form a yellow pigment known as macular pigment (MP) [10]. There is growing body of evidence to suggest that carotenoids L and Z may prevent the development or progression of age-related macular degeneration (AMD) [11–14]. The recently conducted Age-related Eye Disease Study 2 demonstrated protective effect of L plus Z supplementation in patients with AMD, when exploratory subgroup analyses of the treatment effects were limited to participants in the lowest quintile of dietary L plus Z [15].

The carotenoids form part of the exogenous antioxidant system and unlike endogenous antioxidant defense system (glutathione peroxidase) can be modulated using dietary modification and/or nutritional supplementation [16]. Currently, there is no data that have reported the associations between carotenoids and retinal vascular parameters. Understanding such associations will further provide evidence that retinal vascular parameter may reflect oxidative stress in retina and carotenoids may have the potential to prevent such adverse alterations in the retinal vasculature. In this cross-sectional study, we evaluated the association between serum carotenoids and quantitative measures of retinal vascular parameters in elderly subjects from a Singapore Chinese population.

## Materials and methods

In this cross-sectional study, 128 Singaporeans subjects were recruited in the department of Ophthalmology and Visual Sciences at Khoo Teck Puat Hospital over a period of 2 years. Potentially eligible study subjects were identified from the pool of patients (as well as relative accompanying patients) visiting our Eye Clinic and through word of mouth to fellow ophthalmologists. The study was approved by the Research and Ethics Committee of the National Healthcare Group and the research procedures followed the tenets of the Declaration of Helsinki.

All subjects underwent a comprehensive eye examination including slit-lamp biomicroscopy for anterior and posterior segment examination. The inclusion criteria included: age 40 years and above, either gender, Chinese ethnicity, able to give informed consent, best-corrected visual acuity 20/40 and better and absence of anterior and posterior segment diseases. Patients diagnosed with systemic diseases, such as hypertension, diabetes mellitus, ischemic heart disease and stroke, were excluded. Subjects with a history of major psychiatric illness and/or poor cognitive function precluding the giving of informed consent were also excluded from the study.

After an informed written consent, the following details were collected using data collection form: socio-demographic history; life style information; family history; medical history and drug history; anthropometric measurements (height in meters and weight in kilograms); blood sample for serum estimation of carotenoids and lipid profile; posterior segment photograph.

### Measurement of quantitative changes in retinal vascular parameters

A 45 degree colour fundus photograph was taken using digital retinal camera (Canon CR-DGi 10D; Canon, Japan) in each study subject. Following pupil dilation using 1% tropicamide and 2.5% phenylephrine hydrochloride, two retinal photographs of each eye were obtained, one centered at the optic disc and the other centered at the fovea.

We used a semi-automated computer-assisted program (SIVA, software version 3.0) to quantitatively measure the retinal vascular parameters (arteriolar/venular caliber, branching angle, tortuosity and fractal dimension) from retinal photographs. The software employs automatic optic disc detection and measures caliber and geometry of peripheral vessels up to 2 DD (disc diameters) from the optic disc margin. Measurements were taken within a concentric grid (0.5–2.0 DD) centered on the optic disc. The program automatically traced and identified all vessels (artery or vein) within the concentric circular grid, thus generating a skeleton image of the retinal microvasculature.

Trained graders, masked to participant characteristics, were responsible for the visual evaluation of SIVA automated measurement and manual intervention if necessary according to a standardized protocol. The measured area was standardized and defined as the region between 0.5 and 2.0 disc diameters away from the disc margin and all visible vessels coursing through the specified zone were measured. The intra- and inter-grader reliability for the measurement was assessed and reported previously.

#### Retinal vascular caliber

**Fig 1:**
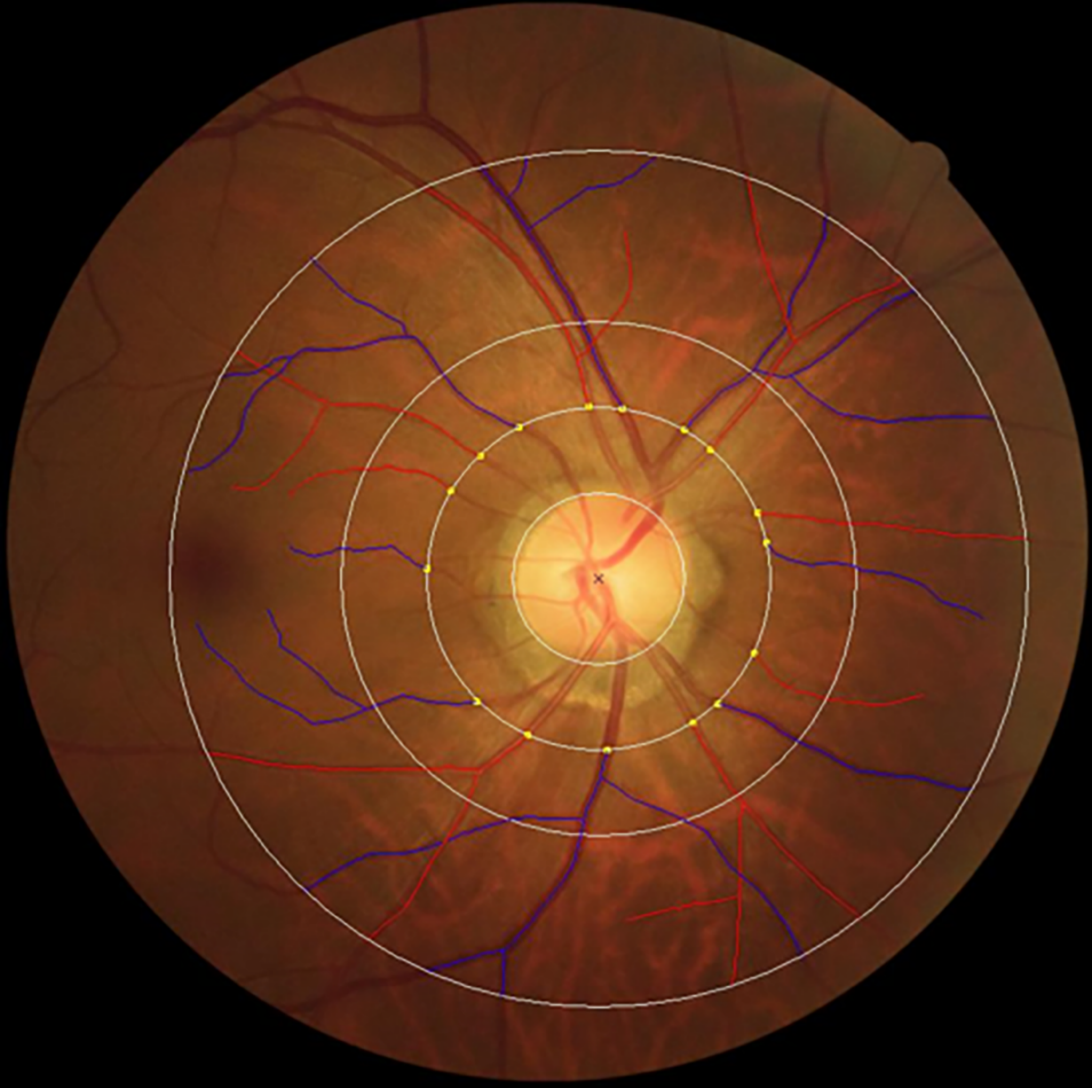
Measurement of retinal vascular calibre using color fundus photographs. SIVA software automatically identified and traced the retinal vessels (arteries and veins) on the digital colour fundus photographs. To further ascertain the accuracy of the automated vessel tracing generated by the software, trained graders examined the traced vessels and made manual corrections as necessary. Central retinal arteriolar and vascular caliber were summarized as CRAE and CRVE, respectively based on the revised Knudtson–Parr–Hubbard Formula.

Retinal vascular caliber was measured that followed the standardized protocol used in the Atherosclerosis Risk in Communities Study based on the revised Knudtson-Parr-Hubbard formula using the SIVA program [17]. The calibers of the central retinal artery and vein were estimated using the ‘Big-6 formula’ and summarized as the central retinal artery and vein equivalents (CRAE and CRVE) representing the average diameter of the arterioles and venules of the eye, respectively (Fig 1) [18].

#### Retinal vascular branching angle

**Fig 2:**
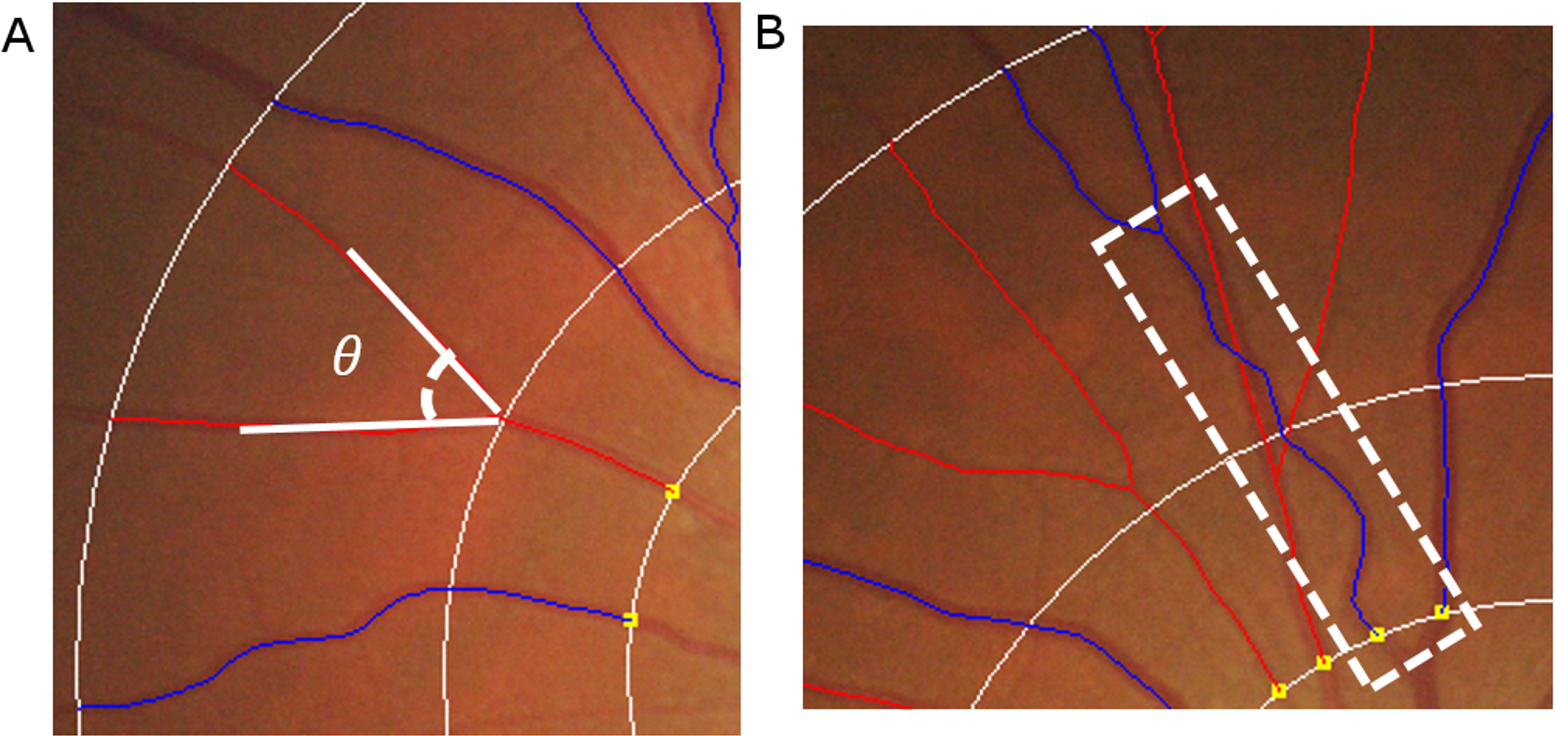
Measurement of retinal branching angles and vascular tortuosity using color fundus photographs. For measurement of retinal branching angle, the lines denoting the direction of the branches were produced by SIVA software that tracked down each vessel in a specified area (defined as the region from 0.5 to 2 disc diameters away from the disc margin). Then the angle subtended by the daughter branches was automatically calculated by the software using the cosine rule of the angle at the bifurcation of the vessels. For assessment of retinal vascular tortuosity, the centerline of the vessel was automatically traced by the SIVA software. The retinal vascular tortuosity was derived from the integral of the curvature square along the path of the vessel, normalized by the total path length measured in a specified area (defined as the region from 0.5 to 2 disc diameters away from the disc margin). A straight vessel has lower tortuosity value while a tortuous vessel has a higher tortuosity value.

Retinal vascular branching angle was defined as the first angle subtended between two daughter vessels at each vascular bifurcation (Fig 2, part A). The retinal vascular branching angle measurement was not bounded by the standardized measured region (within 0.5 and 2.0 disc diameters).

#### Retinal vascular tortuosity

Retinal vascular tortuosity was computed as the integral of the curvature square along the path of the vessel, normalized by the total path length (Fig 2, part B); this measure is dimensionless as it represents a ratio measure. Retinal vascular tortuosity reflects the straightness/curliness of the vessels; a larger tortuosity value indicates a curvy retinal vessel.

#### Retinal vascular fractal dimension

Retinal vascular fractal dimension was calculated from a skeletonized line tracing using the box-counting method, and represents a “global” measure that summarizes the whole branching pattern of the retinal vascular tree (or property of self-similarity). Larger values indicate a more complex branching pattern.

### Serum analysis of carotenoids

Fasting blood sample was collected in two 5ml vacutainer tubes containing 4.5U sodium or lithium heparin per milliliter of whole blood. The samples were centrifuged at 5000 rpm for 10 min within 8–10 hours and the separated layer of serum was then aliquoted into two light sensitive micro centrifuge tubes and stored at -70°C until analysis.

A Hewlett-Packard (HP 1090 LC) system with photodiode array detection at 292, 325 and 450nm was employed, using Agilent Chem Station software. A 5μm analytical/preparative 4.6 x 250mm 201 TP specialty reverse phase column (Vydac) was used with an in-line guard column. The mobile phase consisted of 97% methanol and 3% tetrahydrofuran was degassed using an in-line degasser. The flow rate was 1ml/min. Hoffmann-La Roche provided the standards for high performance liquid chromatography analysis.

A 30μL aliquot of serum was de-proteinized with equal volume of ethanol-tert-butanol 4:1, v/v (EB solution) and internal standard (echinenone, 0.4mg/L) in an amber micro-centrifuge tube. This was extracted with 100μL of n-hexane for 2 min. After centrifugation at 15000r/min, 160μL of supernatant was transferred into another amber microcentrifuge tube and dried under a stream of nitrogen. The dried residue was reconstituted into 60μL of EB solution and 30μL of this solution was used for the analysis. Pure acetonitrile, pure methanol, and a mixture of ethanol and tert-butanol (8:2, v/v) constituted the three mobile phase solutions used for gradient separation. The gradient profile of mobile phase (A:B:C) was set at 75:25:0 from 21-30min using a Waters Alliance 2695 separation module. During the first 8min of analysis, L that co-eluted with Z on column-1 (Agilent Zobrax SB-_18_, 4μm, 150x3.9mm I.D., 25°C) was directed to column-2 (Whatman Partisphere-5C_18_, 5μm, 110x4.7mm, I.D., 4°C). Following the last elution of less-polar compounds from column-1 from 8-24min, the flow was redirected to column-2 to separate L and Z, which eluted from 27-30min. The peak heights were monitored at wavelength 450nm using a PDA detector.

### Assessment of other study variables

Fasting venous blood samples were analyzed at the Khoo Teck Puat Hospital Reference Laboratory for biochemical testing of serum total cholesterol, triglycerides, HDL cholesterol and LDL cholesterol. Current smokers were defined as those currently smoking any number of cigarettes (i.e. current versus past/never). Alcohol consumption was defined as those currently drinking alcoholic beverages daily or on some days (i.e., current versus past/never). Body mass index (BMI) was calculated as body weight (in kilograms) divided by body height (in meters) squared.

### Statistical analysis

All statistical analyses were performed using SPSS statistics version 22.0. Independent t-test was used to compare the characteristics. Quantitative retinal vascular parameters were analyzed as continuous variables (per standard deviation [SD] increase). Linear regression models were used to analyze the association of retinal vascular parameters with serum carotenoids. Multiple linear regression models were adjusted for age, gender, current smoking, BMI, total cholesterol, HDL cholesterol and triglycerides levels. The models testing CRAE or CRVE related to serum carotenoids were additionally adjusted for fellow caliber in order to provide unbiased and biologically plausible results as suggested by Liew et al [19].

## Results

In this study, we evaluated the relationship between serum carotenoids and quantitative measures of retinal vascular parameters in 128 healthy elderly subjects. Although 134 subjects were recruited, 6 subjects were excluded due to the following reasons: insufficient quality of fundus photographs (dense cataract, n = 2; small pupil, n = 1), non availability of blood sample due to technical reason (n = 1) and intake of hypertensive medications (n = 2) although subjects denied history of hypertension. Of the 128 subjects, 12 (9.6%) subjects claimed that they were diagnosed with hyperlipidemia; however, they were not on any medical treatment at the time of recruitment. The serum lipid profile in subjects with hyperlipidemia did not differ significantly from rest of the study population (serum cholesterol: hyperlipidemia = 5.228; no hyperlipidemia = 5.561, p = 0.212).

The mean (± SD) age of the study population was 54.1 (±7.41) years, ranging from 40 to 81 years and there was slight preponderance of female subjects (60%). Table 1 shows the demographic characteristics of the study population stratified by gender.

**Table 1:** Demographic characteristics of study population stratified according to gender.

	Male (n = 51)		Female (n = 77)		
	Mean	SD	Mean	SD	p value
**Age**	55.61	7.49	53.17	7.25	0.068
**BMI**	23.91	3.53	22.77	3.40	0.070
**Cholesterol**	5.34	0.91	5.65	0.84	0.059
**Triglycerides**	1.61	0.88	1.20	0.56	**0.002**
**HDL-C**	1.49	0.50	1.88	0.44	**0.000**
**LDL-C**	3.55	1.03	3.70	0.77	0.359
**C: HDL ratio**	3.87	1.20	3.14	0.73	**0.000**
**Serum L**	0.32	0.19	0.30	0.18	0.668
**Serum Z**	0.09	0.05	0.068	0.03	**0.021**

HDL: High density lipoproteins; LDL: Low density lipoproteins; C: Cholesterol; L: Lutein; Z: Zeaxanthin

Male subjects demonstrated statistically significant higher serum triglycerides (p = 0.002) and cholesterol/HDL ratio (p<0.001) but lower HDL cholesterol (p < 0.001) when compared to females. A relatively higher concentration of serum L and Z was observed in males when compared to females but this relationship reached statistical significance for only serum Z (p = 0.021). 15 (11.7%) subjects gave a positive history of current smoking and all these were males.

A significant and positive relationship was observed between serum concentrations of L and Z (r = 0.452, p <0.001). Table 2 shows the relationship of retinal vascular parameters with serum concentrations of L and Z.

**Table 2:** Relationship between retinal vascular parameters and serum lutein and zeaxanthin.

Retinal vascular parameters			Serum Lutein				Serum Zeaxanthin			
(per SD increase)	Mean	SD	β	95% CI		P value	β	95% CI		P value
Arteriolar Caliber	117.5	9.5	**0.045**	0.003	0.086	**0.036**	0.0001	-0.0087	0.0090	0.977
Venular Caliber	166.5	12.3	**-0.045**	-0.086	-0.003	**0.035**	0.0044	-0.0044	0.0132	0.323
Arteriolar Tortousity	0.944 (x10^4)	0.147 (x10^4)	0.009	-0.026	0.043	0.618	-0.0036	-0.0108	0.0036	0.326
Venular Tortousity	1.18 (x10^4)	0.183 (x10^4)	-0.026	-0.061	0.008	0.126	**-0.0075**	-0.0145	-0.0004	**0.039**
Arteriolar Fractal Dimension	1.244	0.046	0.018	-0.017	0.054	0.313	-0.0020	-0.0095	0.0054	0.589
Venular Fractal Dimension	1.238	0.049	0.003	-0.032	0.039	0.858	-0.0044	-0.0117	0.0029	0.239
Arteriolar Branching Angle	72.98	11.31	**-0.039**	-0.072	-0.006	**0.021**	**-0.0073**	-0.0142	-0.0003	**0.041**
Venular Branching Angle	77.30	10.18	-0.011	-0.045	0.023	0.529	-0.0013	-0.0084	0.0059	0.721

Multiple linear regression models adjusted for age, gender, current smoking, body mass index, total cholesterol, HDL cholesterol and triglycerides

In multiple linear regression analysis, per SD decrease in retinal arteriolar caliber was associated with decreased levels of serum L (β = 0.045 [0.003 to 0.086], p = 0.036) whereas per SD increase in retinal venular caliber (β = -0.045 [-0.086 to -0.003], p = 0.036) was associated with decreased levels of serum L. Per SD increase in retinal venular tortuosity was associated with decreased serum levels of Z (β = -0.0075 [-0.0145 to -0.0004], p = 0.039). Per SD increase in arteriolar branching angle was associated with decreased levels of serum L (β = -0.039 [-0.072 to -0.006], p = 0.021) as well as serum Z (β = -0.0073 [-0.0142 to -0.0059], p = 0.041). Age, gender, current smoking, BMI, total cholesterol, HDL cholesterol and triglycerides levels were included as confounding variables in the multiple linear regression analysis.

**Table 3**shows the relationship of retinal vascular parameters with other serum carotenoids evaluated in the study (all-trans-retinol, cryptoxanthin, carotene, lycopene, tocopherols and tocotrienols) after adjusting for confounding variables. None of these carotenoids demonstrated a meaningful relationship with retinal vascular parameters with the exception of all-trans-retinol and vascular caliber (only one vascular parameter). Per SD decrease in retinal arteriolar caliber was associated with decreased serum levels of all-trans-retinol (β = 0.195 [0.002 to 0.083], p = 0.038) whereas per SD increase in retinal venular caliber was associated with decreased serum levels of all-trans-retinol (β = -0.271 [-0.099 to -0.019], p = 0.004).

**Table 3:** Relationship between retinal vascular parameters and other serum carotenoids.

	CALIBER		TORTOUSITY		FRACTAL DIMENSION		BRANCHING ANGLE	
	Arteriolar	Venular	Arteriolar	Venular	Arteriolar	Venular	Arteriolar	Venular
**All-Trans-Retinol**								
β Value	0.043	0.059*	0.006*	0.023	0.023*	0.015*	0.008*	0.012*
95% Confidence interval	0.002 to 0.083	0.099* to 0.019*	0.040* to 0.027	0.011* to 0.056	0.057* to 0.012	0.050* to 0.019	0.041* to 0.025	0.045* to 0.022
P value	**0.038**	**0.004**	0.718	0.180	0.199	0.377	0.648	0.491
**A-Cryptoxanthene**								
β Value	0.002*	0.002	0.005*	0.003*	0.004	0.002	0.002	0.000*
95% Confidence interval	0.008* to 0.004	0.004* to 0.009	0.010* to 0.000	0.008* to 0.002	0.002* to 0.009	0.003* to 0.007	0.003* to 0.007	0.005* to 0.005
P value	0.517	0.440	**0.040**	0.230	0.169	0.400	0.340	0.986
**B-Cryptoxanthene**								
β Value	0.031*	0.006	0.022*	0.007*	0.019	0.029	0.006*	0.017*
95% Confidence interval	0.081* to 0.019	0.043* to 0.056	0.063* to 0.018	0.048* to 0.033	0.023* to 0.061	0.012* to 0.070	0.046* to 0.033	0.057* to 0.023
P value	0.221	0.799	0.269	0.723	0.377	0.162	0.757	0.401
**A-Carotene**								
β Value	0.005	0.015*	0.003*	0.001	0.007	0.007	0.003*	0.000
95% Confidence interval	0.007* to 0.018	0.028* to 0.003*	0.013* to 0.007	0.009* to 0.011	0.003* to 0.018	0.003* to 0.018	0.013* to 0.007	0.011* to 0.010
P value	0.420	**0.015**	0.535	0.850	0.175	0.182	0.547	0.950
**B-Carotene**								
β Value	0.004*	0.060*	0.022*	0.005	0.024	0.26	0.024*	0.047*
95% Confidence interval	0.080* to 0.071	0.135* to 0.015	0.083* to 0.040	0.057* to 0.067	0.041* to 0.088	0.038* to 0.089	0.084* to 0.037	0.108* to 0.014
P value	0.911	0.118	0.486	0.879	0.469	0.424	0.441	0.132
**Lycopene**								
β Value	0.003*	0.008	0.005*	0.004*	0.004*	0.005*	0.003	0.004
95% Confidence interval	0.013* to 0.007	0.002* to 0.019	0.013* to 0.003	0.012* to 0.004	0.012* to 0.005	0.014* to 0.003	0.005* to 0.011	0.005* to 0.012
P value	0.544	0.099	0.201	0.311	0.409	0.231	0.402	0.396
**A-Tocopherols**								
β Value	0.276*	0.556	0.020*	0.356*	0.251*	0.273*	0.046	0.649*
95% Confidence interval	0.781* to 0.230	0.051 to 1.062	0.431* to 0.395	0.766* to 0.055	0.682* to 0.179	0.698* to 0.152	0.454* to 0.361	1.046* to 0.252*
P value	0.282	**0.031**	0.926	0.089	0.250	0.205	0.822	**0.022**
**D-Tocopherols**								
β Value	0.003*	0.008	0.001*	0.008*	0.000	0.002*	0.004	0.000
95% Confidence interval	0.011* to 0.005	0.001* to 0.016	0.008* to 0.005	0.014* to 0.001*	0.007* to 0.007	0.009* to 0.005	0.003* to 0.010	0.007* to 0.007
P value	0.474	0.069	0.686	**0.026**	0.972	0.597	0.291	0.976
**G-Tocopherols**								
β Value	0.010*	0.019	0.037*	0.028*	0.000	0.026*	0.023*	0.000
95% Confidence interval	0.060* to 0.040	0.031* to 0.068	0.076* to 0.003	0.068* to 0.012	0.042* to 0.042	0.067* to 0.015	0.062* to 0.016	0.040* to 0.041
P value	0.699	0.463	0.071	0.171	0.991	0.210	0.250	0.986
**A-Tocotrienol**								
β Value	0.000	0.000	0.001	0.000	0.000	0.001*	0.000	0.000
95% Confidence interval	0.002* to 0.001	0.002* to 0.002	0.001* to 0.002	0.002* to 0.001	0.002* to 0.001	0.002* to 0.001	0.001* to 0.001	0.002* to 0.001
P value	0.802	0.925	0.434	0.595	0.709	0.425	0.741	0.729
**D-Tocotrienol**								
β Value	0.001*	0.013*	0.010	0.006	0.009	0.009	0.001	0.007*
95% Confidence interval	0.0118 to 0.010	0.023* to 0.002*	0.001 to 0.019	0.003* to 0.015	0.000 to 0.018	0.000 to 0.018	0.008* to 0.010	0.016* to 0.002
P value	0.902	**0.018**	**0.023**	0.170	0.061	0.058	0.791	0.112
**G-Tocotrienol**								
β Value	0.000	0.001*	0.000	0.001	0.000	0.000	0.000	0.001*
95% Confidence interval	0.002* to 0.002	0.003* to 0.001	0.001* to 0.002	0.001* to 0.003	0.001* to 0.002	0.001* to 0.002	0.002* to 0.002	0.003* to 0.001
P value	0.861	0.364	0.669	0.383	0.700	0.652	0.891	0.234

Multiple linear regression models adjusted for age, gender, current smoking, body mass index, total cholesterol, HDL cholesterol and triglycerides*Negative correlation

## Discussion

In this cross-sectional study, we evaluated the association between retinal vascular parameters and serum carotenoids in healthy elderly subjects. We found that measures of adverse retinal vasculature (e.g. narrow arteriolar caliber, wide venular caliber, venular tortuosity, and wide arteriolar branching angle) were associated with lower serum levels of carotenoids L and Z.

This is the first study to observe an association between quantitative measures of retinal vascular parameters and serum L and Z. Of note, none of the other serum carotenoids explored in this study demonstrated meaningful relationship with more than one retinal vascular parameter. We believe that these observations are unsurprising given that the primate retina uniquely concentrates L and Z, to the exclusion of all other dietary carotenoids, accounting for 80–90% of these carotenoids in the human retina [20]. Carotenoids, L and Z, by nature of their biochemical structure and function, help neutralize reactive oxygen species, and thereby, prevent oxidative stress in the retina [21, 22]. L and Z constitute part of exogenous antioxidant defense system because they cannot be synthesized *in-vivo* in the human body and are derived entirely from the dietary sources of these carotenoids [23].

Currently there are few comparative studies available. Daien et al. demonstrated that narrower retinal arteriolar caliber was independently associated with lower glutathione peroxidase activity.^8^ Glutathione peroxidase constitutes endogenous antioxidant defense system and plays a role in the regulation of redox state of vascular cells [24]. Of note, only one retinal vascular parameter was measured in that study (i.e. vascular caliber). Similarly, Kan et al. observed significant association between lower intake of dietary fiber and retinal vascular caliber in the form of narrower arteriolar caliber and wider venular caliber, after adjustment of potential confounders [25]. Investigators proposed that participants with lower fiber intake have generally lower intake of carotenoids (p <0.001) along with other micronutrients that may have partly contributed to the observed associations.

The underlying mechanisms for the association between serum L and Z and retinal vascular caliber remain unclear. Steinberg et al. hypothesized that LDL that has undergone oxidative damage is considerably more atherogenic that native LDL [26]. LDL oxidation involving reactive oxygen species takes place in vivo and contributes to the clinical manifestation of atherosclerosis supports oxidative modification hypothesis [27]. We speculate that circulating L and Z may decrease oxidation of lipids within the LDL molecule via its antioxidant properties. This leads to reduced uptake of oxidized lipids by macrophages and its deposition within the intimal layers of the retinal vessels thereby reducing retinal arteriosclerosis and its effect on retinal vascular caliber.

In the eye, the RPE is a highly selective transfer point from the choroidal vessels where lipoprotein-bound L and Z must cross to reach the photoreceptors of the retina. Inter photoreceptor retinoid binding protein may facilitate transport of L and Z to retinal cells by CD36 but the specificity and uptake are ultimately driven by selective bindings proteins [28]. When not bound to proteins, L and Z easily insert themselves into biological membranes and have been shown to increase the rigidity of the lipid bilayer where they can act as “molecular rivets” because of the orientation within the membrane [29]. The effect of L and Z on lipid membranes’ structural and dynamic properties seems to decrease the lipid bilayers susceptibility to oxidative degradation [30].

A similar protective role of carotenoids based on the ‘oxidative hypothesis of atherogenesis” has also been proposed in clinical and subclinical cardiovascular diseases [31]. Several lines of evidence suggest that LDL must be oxidatively modified to trigger the pathological event of retinal arteriosclerosis [32, 33]. Furthermore, findings from experimental [34] and population based studies (The Rotterdam Study, Los Angeles Atherosclerosis Study and the Beijing atherosclerosis study) [35–37] have suggested a protective role of the carotenoids in the development and/or progression of systemic atherosclerosis. Systemic atherosclerosis in these studies was assessed using calcified plaques of the aorta or intima-media thickness of the common carotid artery.

Alternatively, carotenoids L and Z may modulate the arteriosclerosis process in retinal vessels by varying the expression of genes involved with inflammation. The reason is that alteration in retinal vascular caliber (arteriolar narrowing and venular widening) may reflect early microvascular pathology, such as inflammation [38]. And, oxidative stress is considered a strong stimulus for activation of a number of pro-inflammatory pathways. Bian et al. have shown that antioxidants L and Z may prevent increase in retinal redox-sensitive NF-kB, which controls the expression of many inflammation-related genes [39]. L via its antioxidant property may block the activation of NF-kB, which plays important role in many pathological reactions and degradation of the inhibitor kB (I-kB) [40, 41]. NF-kB translocates into the nucleus, upon dissociation of I-kB from the NF-kB complex by L, decreasing inducible gene transcription and synthesis of inflammatory markers [42].

In addition, carotenoid Z may play a role in the regulation of the redox state of the vascular cells that may potentially impact the retinal microcirculation (vascular caliber). Following supplementation with Z, there was decreased levels of oxidized glutathione and increased levels of reduced glutathione (intracellular) as well as increase in the ratio of reduced/oxidized glutathione in response to oxidative stress [43]. Glutathione is a powerful endogenous antioxidant that protects cells from oxidative stress and can be generated from oxidized glutathione [44]. Therefore, it appears that Z exhibits antioxidant function at least partly by regulating the synthesis and levels of glutathione. As a consequence, there is improvement in intracellular redox status along with reduction in the susceptibility to free radical induced cell death (hydrogen peroxide) [45]. This observation also strengthens the fact that endogenous and exogenous antioxidant defense systems may work in tandem.

Furthermore, we also observed increase retinal venular tortuosity in association with lower serum levels of Z. Increase in vessel tortuosity as evidenced by increase in tortuosity values is the first manifestation of the changes in vessel morphology. Venules exhibit different optimal flow characteristics across the vascular network compared with arterioles due to different physiological function [46]. An increase in venular tortuosity is associated with pressure drop and decrease in blood velocity leading to sluggish blood flow [47]. Indeed, an increase in venular tortuosity has been associated with systemic (such as hypertension and ischemic heart disease mortality) [48,49] as well as ocular diseases (such as diabetic retinopathy and central retinal vein occlusion) [50,51].

Lastly, we also demonstrated an association of larger arteriolar branching angle with lower serum levels of L as well as Z. The geometry of arterioles may affect the efficiency of circulation i.e. the ability of the arteriolar tree to deliver blood to tissue with a minimum total blood volume with minimum loss of energy at each branching angle [52]. Efficiency in blood flow is reduced when branching angle is deviated from the optimal retinal vascular network [53]. Also, Chapman et al demonstrated that branching angle was likely to increase in response to less oxygen saturation [54]. Therefore, larger branching angle in association with lower levels of carotenoids L and Z suggest that these carotenoids may maintain optimum oxygen saturation by preventing alteration in the retinal vascular geometry.

There are several strengths of this study that are worth mentioning. This is the first study to examine the relationship between serum carotenoids and quantitative measures of retinal vascular parameters. Subjects with only Chinese ethnicity were recruited since ethnicity may affect retinal vascular parameters. Also, fasting samples were collected for serum analysis of carotenoids and mean of duplicate extractions were used for the purpose of analysis. Furthermore, quantitative assessment of retinal vascular parameters was performed by a robust and reliable technique and evaluations were conducted by a single observer to eliminate the inter-observer variability of measurements and reduce sources of bias. Lastly, being non-dimensional, retinal vascular parameters are less likely to be affected by variations in digital image resolution, ocular magnification or differences in refractive errors.

There are several limitations in this study. First and foremost is that it is a cross-sectional study, therefore, a causal and temporal relationship between serum L and Z and retinal vascular parameters cannot be established. Second, we used single blood specimen to determine serum carotenoids. Given the biologic variation of the actual carotenoid concentration, a single serum specimen may not be able to provide a precise estimate of typical serum carotenoid concentrations over time. MP levels would have been helpful since it correlates with serum L and Z, can be measured non-invasively, and is less variable, however, we did not include MP measurements in this study. Third, despite the standardized protocol used, the quantitative measurement of retinal vascular grading (particularly tortuosity) may include measurement errors related to subjective grader input, variability in image quality and other unknown issues (e.g. algorithmic procedures, pulse cycle) that may lead to less precision of the measurement. Lastly, there may be residual confounding factors that we have not controlled for and that could have biased or modified the associations observed in our study.

In conclusion, lower levels of L and Z demonstrated significant relationship with adverse quantitative measures of retinal vasculature in healthy elderly subjects from a Singaporean Chinese population. This suggests that antioxidant carotenoids L and Z may allow optimal geometry of the retinal microcirculation to maintain efficient blood flow and to prevent the onset of subclinical vascular diseases. However, prospective large scale studies are warranted to confirm our observations in the near future.

## Supporting information

S1 Table Supporting Information Files. (XLSX)
